# Estructura de comunidades en las redes semánticas de la investigación biomédica sobre disparidades en salud y sexismo

**DOI:** 10.7705/biomedica.5182

**Published:** 2020-12-11

**Authors:** Lucero Soledad Rivera-Romano, Gabriela Juárez-Cano, Enrique Hernández-Lemus, Maite Vallejo, Mireya Martínez-García

**Affiliations:** 1 Programa de Licenciatura en Promoción de la Salud, Universidad Autónoma de la Ciudad de México, Ciudad de México, México Universidad de la Ciudad de México Universidad Autónoma de la Ciudad de México México Mexico; 2 División de Genómica Computacional, Instituto Nacional de Medicina Genómica, Ciudad de México, México Instituto Nacional de Medicina Genómica México México; 3 Departamento de Investigación Sociomédica, Instituto Nacional de Cardiología Ignacio Chávez, Ciudad de México, México Instituto Nacional de Cardiología Ignacio Chávez México México

**Keywords:** investigación biomédica, calidad de la atención de salud, disparidades en el estado de salud, sexismo, minería de datos, interpretación estadística de datos, web semántica, Biomedical research, quality of health care, health status disparities, sexism, data mining, data interpretation, statistical, semantic web

## Abstract

**Introducción.:**

Como una iniciativa para mejorar la calidad de la atención sanitaria, en la investigación biomédica se ha incrementado la tendencia centrada en el estudio de las disparidades en salud y sexismo.

**Objetivo.:**

Caracterizar la evidencia científica sobre la disparidad en salud definida como la brecha existente entre la distribución de la salud y el posible sesgo por sexo en el acceso a los servicios médicos.

**Materiales y métodos.:**

Se hizo una búsqueda simultánea de la literatura científica en la base de datos Medline PubMed de dos descriptores fundamentales: *Healthcare disparities* y *Sexism.* Posteriormente, se construyó una red semántica principal y se determinaron algunas subunidades estructurales (comunidades) para el análisis de los patrones de organización de la información. Se utilizó el programa de código abierto Cytoscape para el analisis y la visualización de las redes y el MapEquation, para la detección de comunidades. Asimismo, se desarrolló código ex profeso disponible en un repositorio de acceso público.

**Resultados.:**

El corpus de la red principal mostró que los términos sobre las enfermedades del corazón fueron los descriptores de condiciones médicas más concurrentes. A partir de las subunidades estructurales, se determinaron los patrones de información relacionada con las políticas públicas, los servicios de salud, los factores sociales determinantes y los factores de riesgo, pero con cierta tendencia a mantenerse indirectamente conectados con los nodos relacionados con condiciones médicas.

**Conclusiones.:**

La evidencia científica indica que la disparidad por sexo sí importa para la calidad de la atención de muchas enfermedades, especialmente aquellas relacionadas con el sistema circulatorio. Sin embargo, aún se percibe un distanciamiento entre los factores médicos y los sociales que dan lugar a las posibles disparidades por sexo.

## Abstract (en)

**Introduction:**

As an initiative to improve the quality of health care, the trend in biomedical research focused on health disparities and sex has increased.

**Objective:**

To carry out a characterization of the scientific evidence on health disparity defined as the gap between the distribution of health and the possible gender bias for access to medical services.

**Materials and methods:**

We conducted a simultaneous search of two fundamental descriptors in the scientific literature in the Medline PubMed database: healthcare disparities and sexism. Subsequently, a main semantic network was built and some structural subunits (communities) were identified for the analysis of information organization patterns. We used open-source software: Cytoscape to analyze and visualize the semantic network, and MapEquation for community detection, as well as an ad hoc code available in a public access repository.

**Results:**

The core network corpus showed that the terms on heart disease were the most common among the descriptors of medical conditions. Patterns of information related to public policies, health services, social determinants, and risk factors were identified from the structural subunits, but with a certain tendency to remain indirectly connected to the nodes of medical conditions.

**Conclusions:**

Scientific evidence indicates that gender disparity does matter for the care quality in many diseases, especially those related to the circulatory system. However, there is still a gap between the medical and social factors that give rise to possible disparities by sex.

La reducción de las brechas en el conocimiento científico relacionadas con las disparidades por sexo y la equidad en salud, debe ser una prioridad para mejorar la calidad de la atención médica. En la literatura biomédica se ha venido incrementando la comprensión de que el sexo sí importa en el proceso de salud-enfermedad y para la calidad de la atención médica, la cual puede verse afectada desde la perspectiva biológica y por el contexto social ^1^. La política y los programas de salud global a menudo son ciegos a las diferencias entre sexos y, sobre todo, a la posición desigual de las mujeres en la sociedad, lo que hace que estas sean "visiblemente invisibles" ^2^.

Para comenzar, es pertinente definir conceptualmente el sexo y el género para luego abordar las disparidades en salud. Por *sexo* se entiende el conjunto de influencias biológicas, es decir, las diferencias reproductivas, hormonales y genéticas entre mujeres y hombres. El *género,* en cambio, alude a los roles, comportamientos, actividades y atributos socialmente construidos que una sociedad considera apropiados para hombres y mujeres ^3^. Para fines de esta investigación, se utilizará el concepto de sexo en el análisis semántico de la información biomédica y el concepto de disparidades en salud según la definición de la *National Library of Medicine of the United States* (Medline) PubMed: diferencias en el acceso o disponibilidad de servicios médicos.

El sexo es una característica biológica determinante para la salud y documentar ese efecto es un punto de partida obligado para la investigación de las disparidades como forma de contribuir al desarrollo de intervenciones diseñadas para reducirlas ^4^. La inclusión sexual es una cuestión de integridad científica. La brecha en la representación de las mujeres en los estudios de ciencia básica ha sido bien documentada y, hasta hace poco, no se había hecho mucho por mitigarla ^5^^-^^8^.

Este hecho ha sido acertadamente abordado en las directrices del reporte *The Sex and Gender Equity in Research* (SAGER), una guía para reportar los resultados de los estudios biomédicos por sexo y género, incorporando esta dimensión desde la planificación y el diseño del estudio, el análisis de los datos y la interpretación de los resultados, con el fin de promover la equidad, la ética, la responsabilidad social y un mayor aprovechamiento de la evidencia científica ^9^. Por su parte, la Organización Mundial de la Salud (OMS) ha determinado brechas de conocimiento sustanciales en el estudio de la interacción del sexo con otros factores sociales determinantes de la salud, recomendando que los avances en investigación biomédica no se desarticulen de tales factores ^5^.

Por otro lado, el marco conceptual en el que se encuadra la disparidad en salud mediada por el sexo se relaciona estrechamente con los factores sociales determinantes, ya sea en su configuración estructural (políticas intersectoriales, gobernanza socioeconómica) o mediadora (empoderamiento e inclusión en los sistemas establecidos para mantener la salud), o en ambas, pues sus efectos muchas veces inciden en la distribución desigual de recursos, lo cual genera la estratificación social y las divisiones de clase que contribuyen a modelar las principales inequidades sociales y afectan el estado de salud de hombres y mujeres ^10^^,^^11^.

Si bien en las últimas décadas muchos países lograron sensibilizarse y mejorar las condiciones generales de salud, lamentablemente los beneficios de estas acciones no se han apreciado en algunos grupos poblacionales, como en el caso de las mujeres. ¿Por qué? Una suposición es que, a pesar de haber muchos estudios científicos, los datos no son reportados de forma desagregada por sexo y eso distorsiona el efecto en los resultados, por ejemplo, en cuanto al reporte de los factores sociales determinantes o de las disparidades en salud, o el tipo y grado de enfermedad o muerte ^12^^-^^14^.

Otra hipótesis es la discrepancia entre la teoría y la práctica en la salud pública, ámbito en el que no se han logrado vincular por completo los conocimientos del paradigma biomédico (muchas veces individualista) y los factores sociales determinantes que subyacen tras el proceso de salud-enfermedad, y que influyen en las elecciones, los conocimientos y los comportamientos de las personas y, por lo tanto, en sus resultados de salud ^11^^,^^15^.

La *medicina basada en el sexo* es una rama innovadora de la investigación biomédica y representa una nueva perspectiva para la promoción de la salud y la calidad de la atención médica ^16^. Sin embargo, con el continuo aumento en la cantidad de literatura, es difícil para los responsables de las decisiones, los médicos y los investigadores utilizar los recursos disponibles y mantenerse al día de manera rápida y efectiva en este tema ^17^.

Aunque los sistemas electrónicos de recuperación de información proporcionan documentos potencialmente útiles, no suelen ayudar a administrar y analizar el gran volumen de información recuperada ^18^. Recientemente se han propuesto diversas metodologías automatizadas para extraer la información de Medline PubMed y visualizarla como un grafo utilizando los términos del vocabulario controlado de los *Medical Subject Headings* (MeSH), términos que se representan como nodos, en tanto que la información compartida entre ellos se representa como enlaces. Este es un método novedoso de abstracción semántica para resumir múltiples textos biomédicos ^19^^,^^20^.

El modelo conexionista se ha empleado justamente para explorar las representaciones léxicas mediante las redes semánticas ^21^. Estas redes permiten la extracción de ideas significativas al establecer grupos de conceptos emergentes o desconocidos de manera inductiva ^22^. El análisis de redes revela propiedades de agrupamiento que proporcionan información sobre la estructura del léxico y la semántica, ninguno de los cuales es directamente observable a partir de los datos aislados ^23^.

El problema de investigación que nos ocupa en este estudio se basa en la conceptualización de la disparidad en salud definida como la brecha existente entre la distribución de la salud y el posible sesgo por sexo de la evidencia científica reportada, en dos sentidos: 1) aquel relacionado con los mecanismos sociales y el acceso oportuno a los sistemas de salud, muchos de ellos evitables e injustos, y 2) el relacionado con los mecanismos biológicos y el manejo médico y clínico, algunos de los cuales serían inevitables, pero que están igualmente vinculados a las diferentes necesidades específicas de hombres y mujeres.

La pregunta planteada en este estudio mediante el uso de redes semánticas es si la información reportada en un corpus de literatura biomédica aborda el tema de la disparidad desagregada por sexo, y si los MeSH sobre temas biomédicos se encuentran vinculados con aquellos sobre factores sociales determinantes relacionados con el acceso y la atención de los servicios de salud.

Con ayuda de la minería computacional de la literatura biomédica y la teoría de grafos, el objetivo del presente estudio fue caracterizar la evidencia científica más reciente sobre las disparidades en salud y el sexismo para, posteriormente, estudiar la estructura de 'comunidades' (o módulos) dentro de las redes semánticas y generar evidencia sobre la representación acusadamente expresiva de los principales descriptores relacionados con los patrones de información en el campo de la investigación biomédica.

## Materiales y métodos

**Figura 1:**
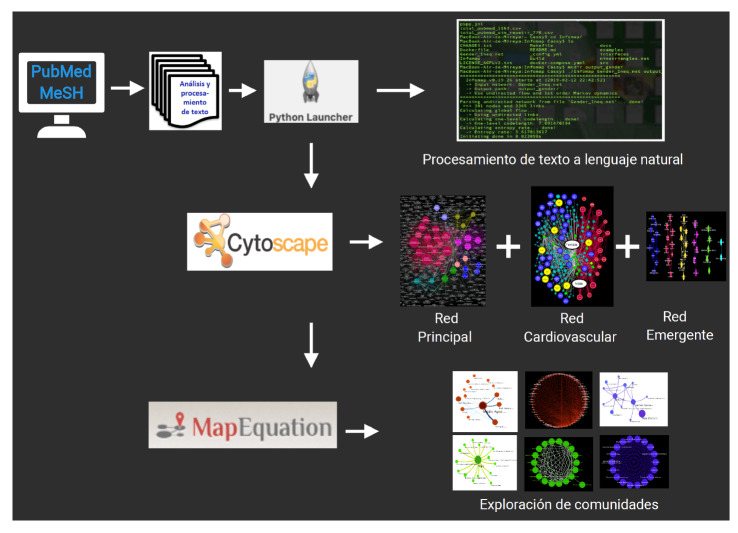
Flujo de trabajo: una vez terminada la minería computarizada de la literatura, se transformó el corpus de información de texto a lenguaje natural. Posteriormente, se construyeron los mapas de conectividad y las redes semánticas y, por último, se utilizó un algoritmo de agrupación para detectar módulos o comunidades y completar el análisis.

La figura 1 resume los pasos seguidos en la investigación con base en la aplicación de las dos estrategias de consulta bibliográfica. Para el análisis de redes basado en los términos MeSH, el 24 de octubre de 2018 se abordó la literatura científica de la base de datos de Medline PubMed mediante minería computarizada empleando simultáneamente dos descriptores sin ninguna restricción: *Healthcare disparities* y *Sexism.* Por otro lado, se consultaron otros documentos de apoyo para contextualizar el problema planteado y reforzar la discusión.

Si bien PubMed es la mayor base de datos de literatura biomédica del mundo, tiene algunas limitaciones. Por ejemplo, cuando se le solicita la recuperación de las citas consultadas para archivarlas, el registro que entrega (por ejemplo en el formato Medline para programas de generación de bibliografías) no incluye todos los autores que se mencionan en los documentos indexados, y también se omite su afiliación institucional y el país. Por otro lado, aunque PubMed contiene más de 15 millones de referencias bibliográficas provenientes de más de 4.000 revistas internacionales, omite aquellas que no se encuentran indexadas en esta base y que podrían contener evidencia científica relevante para las ciencias de la salud.

### Minería computarizada de términos MeSH

Los términos MeSH fueron diseñados por un panel de expertos internacionales en un proyecto dirigido por la *National Library of Medicine* de los Estados Unidos como una ontología, lo que tiene importantes consecuencias cuando se usa como sistema de clasificación hemerobibliográfica y para el procesamiento de la minería de textos asistido por computadora. Una *ontología* es, como se sabe, un sistema conceptual informal basado en una teoría lógica, es decir, vocabulario controlado (finito) en el marco de una teoría lógica.

La ontología MeSH contiene términos semánticamente relacionados, estructurados jerárquicamente para facilitar la consulta y el análisis computacional de las publicaciones incluidas ^24^. Cuenta con más de 57.000 descriptores (títulos principales) y más de 80 calificadores (subtítulos temáticos), cada publicación indexada puede contener uno o varios MeSH que son asignados según el contexto del documento ^25^.

Esto resulta relevante, ya que un vocabulario controlado evita los sesgos de lenguaje debidos, por ejemplo, a la sinonimia o, más complejo aún, la terminología dependiente del contexto. Por ejemplo, para referirse a un crecimiento anormal de tejido debido al cáncer, algunos autores utilizarían la palabra clave "tumor", otros "neoplasia", "cáncer", "nódulo maligno", etc. En todos estos casos, la clasificación ontológica de los MeSH utilizaría "neoplasms" como clasificador, evitando así las ambigüedades.

Aunque las técnicas modernas de procesamiento computacional del lenguaje natural permiten la generación de diccionarios para desambiguar términos similares, todavía son muy proclives a fallar, especialmente cuando se trata de léxicos muy técnicos como puede ser el caso aquí, e introducir sesgos.

Por tanto, la concurrencia de términos MeSH es un recurso muy apreciado para la identificación y la recuperación rápida, precisa y confiable de información ^24^^,^^25^. Por estas razones, se utilizaron los términos MeSH como referencia bibliométrica para construir un mapa de conectividad y redes semánticas en las que, por lo menos, se registraran dos MeSH conectados mediante uno o más *PubMed Unique Identif'ars* (PMID).

### Estrategia de búsqueda de términos MeSH

Para cumplir con el objetivo del estudio, se combinaron dos términos específicos, *Healthcare disparities* y *Sexism,* en la búsqueda de MEDLINE PubMed. Aunque la búsqueda de un solo término MeSH arroja una gran cantidad de resultados, es posible que queden incluidos temas menos específicos. Por otro lado, el uso de otros criterios de búsqueda, como "Title/abstract" o "keywords" puede elevar el número de publicaciones. Sin embargo, la mayoría de las veces este método ya no es necesario, pues el uso combinado de ciertos MeSH permite agrupar la evidencia necesaria y cumplir con el objetivo de la búsqueda, como en este caso.

### Estrategia de selección de los documentos recuperados

Los criterios de selección aplicados en la recuperación de los documentos fueron los siguientes.

*Criterios de inclusión.* Cada registro bibliográfico debía contener, como mínimo, un término MeSH para establecer la conexión en la red de cada documento; el registro bibliográfico podía ser de cualquier año de publicación, cualquier país de adscripción, y cualquier tipo de literatura científica; el término MeSH podía estar en cualquier idioma, indexado en cualquier nivel de la estructura jerárquica de anotación, e incluido en cualquiera de las categorías biomédicas de Medline PubMed.

*Criterios de exclusión.* Se excluyeron aquellos registros que no contenían título o resumen o cuyo contenido no fuera pertinente para el problema en estudio.

### Estrategia de curaduría de la información

Se hizo un curaduría mediante el escrutinio manual de los términos MeSH recuperados que serían conectados en la red para detectar, y en lo posible eliminar, los términos redundantes y que no aportaran información relevante a la búsqueda por sí mismos, lo que solo sucedió con el MeSH *Humans* (común a todos los registros de investigación en salud en humanos y, por lo tanto, redundante), el cual se eliminó, de hecho, antes de analizar la estructura y la conectividad de la red. Posteriormente, el corpus de información se transformó de texto simple a lenguaje natural utilizando un código en el lenguaje de programación Python previamente desarrollado para la construcción de redes semánticas ^5^.

### Construcción de redes semánticas

Las redes semánticas son representaciones gráficas de conocimiento basadas en relaciones significativas de ideas o conceptos que se estructuran como una red de palabras relacionadas cognitivamente entre sí. Se utilizan comúnmente para inferir los marcos teóricos o conceptuales utilizados en los textos y muchas veces su análisis contribuye a que surjan nuevos enfoques a partir de los datos explorados ^18^.

La construcción de redes semánticas se basa en la teoría conexionista de redes complejas. Una *red* es un conjunto de nodos, elementos o vértices que se conectan mediante aristas o enlaces que pueden ser dirigidos, es decir, siguen una dirección de un nodo a otro, o no la siguen. El objetivo del estudio de redes es encontrar las propiedades estadísticas o topológicas que caracterizan su estructura.

A continuación, se describen brevemente algunos de los atributos topológicos o medidas de centralidad utilizados en el presente trabajo.

*Centralidad de vector propio:* mide la influencia de un nodo dentro de una red con base en su importancia relativa frente a otros.

*Distribución de grado:* es la característica más simple que puede observarse en una red, es decir, la conectividad entre nodos adyacentes.

*Coeficiente de agrupamiento:* estima la tendencia de agrupamiento de los nodos en una red; es la probabilidad de que dos nodos puedan estar conectados por un tercer nodo y exista mayor cohesión.

*Promedio de caminos* más corto*s:* indica la longitud promedio del camino más corto entre dos nodos.

*Centralidad de intermediación:* es el número de caminos cortos entre dos nodos que pasan a través de otro.

Aunque hay diferentes atributos para determinar los patrones de conectividad de una red, en este estudio se eligieron los parámetros más simples de la teoría de redes para estimar la conexión de los conceptos, como es el caso de la centralidad de grado, el número total de enlaces de un nodo o la suma de las frecuencias o pesos de las interacciones ^26^. Los nodos con elevada centralidad de grado tienen muchos vecinos y, generalmente, son más frecuentes en la red que los de menor grado ^27^^,^^28^.

Con el fin de discutir similitudes y diferencias con otras medidas de centralidad relevantes, se incluyó un cuadro suplementario (cuadro suplementario 1) con la distribución de las diversas centralidades de los MeSH correspondientes a aquellas enfermedades de interés especial para la discusión posterior. Para visualizar mejor algunos MeSH relacionados con las principales enfermedades encontradas en el corpus de la red, los nodos se marcaron con diferentes colores y tamaños según el número de conexiones con sus principales vecinos. Para analizar y visualizar la red, se utilizó el programa de código abierto Cytoscape ^29^.

### Red principal

Una vez hecha la minería computarizada de la literatura, se construyó una red basada en taxonomías predeterminadas (los términos MeSH), no dirigida ni ponderada. El nodo es la unidad de análisis de la red representada en un mapa de conectividad. Los mapas de conectividad se construyeron de modo que los nodos de origen y destino fueran los MeSH (que representan los conceptos bajo análisis) correspondientes al corpus de los artículos científicos.

Cuando, por lo menos, dos artículos detectados con un número único (PMID) compartían ambos MeSH, se trazaba un enlace (arista) entre estos nodos. Los enlaces utilizados en la construcción de la red fueron los PMID de cada publicación. Este enfoque produjo una red que no consideró el peso relativo de las conexiones. Como se discutirá más adelante, dada la distribución de pesos en las redes resultantes, prácticamente idéntica para cada enlace con pocas excepciones (cuadros suplementarios 1 y 2), este hecho no afectó de manera significativa los resultados o conclusiones del estudio.

### Red secundaria de enfermedad cardiovascular

Como ejemplo para explorar los patrones de conexión de información en una red secundaria, la principal se dividió usando los términos MeSH relacionados con la enfermedad cardiovascular. Se colorearon en rojo aquellos nodos y enlaces asociados con dicha enfermedad, en amarillo, algunos términos sugerentes de factores sociales determinantes; en azul oscuro, los relacionados con los servicios de salud; en verde, aquellos que evocaban las condiciones biológicas y, en azul claro, los términos alusivos a otros factores de estilos y modos de vida.

### Red emergente de la diferencia entre mujeres y hombres

Para inferir posibles patrones de conexión de la información a partir del sexo, también se construyeron redes secundarias de hombres y mujeres, y para garantizar que se pudieran comparar, se filtraron en el programa Cytoscape seleccionando el MeSH relativo al sexo *("female"* o *"male")* en todos sus primeros vecinos. A partir de estas redes secundarias, se diferenciaron sus nodos para conocer los términos incluidos en una y no en la otra, y a la red resultante de esta diferencia se le denominó red emergente.

### Modularidad

La modularidad permite simplificar y resaltar propiedades estructurales de una red para generar subunidades fuertemente interconectadas, conocidas como módulos o comunidades, que contienen un número mayor de enlaces entre nodos que los nodos fuera de ella ^30^^,^^31^.

Hay diversas técnicas de detección de comunidades, pero no hay un método de optimización de la partición modular que funcione en todos los casos ^32^. En este estudio, se utilizó un algoritmo de agrupación de comunidades basado en las caminatas aleatorias y en la teoría de la información centrada en la interdependencia de los enlaces (Infomap). Los módulos de estas subunidades se visualizaron con la aplicación en línea MapEquation ^33^^,^^34^.

Infomap se basa en la dinámica del flujo de información en la red más que en su estructura topológica simple. La MapEquation captura el flujo de información en la red de manera que las comunidades están conformadas por grupos de nodos entre los cuales el flujo de información persiste. A grandes rasgos, para describir el flujo de información en la red, se recurre a una caminata aleatoria que registra dicho flujo (como aproximación). Por ergodicidad, asintóticamente se llega a una distribución estacionaria cuando se visitan estructuras de la red y cuando se las abandona, estructuras que pueden ser nodos o módulos. Estas distribuciones estacionarias se pueden codificar de forma óptima usando la llamada codificación de Huffman en una cadena binaria. Así, la caminata puede describirse en términos de la teoría de la información a partir de la longitud de la cadena binaría asociada con ella ^31^^,^^32^^,^^35^.

Si se tuviera una partición de la red en *M* en módulos, se podría obtener la descripción de una caminata aleatoria entre los módulos (o estructuras más relevantes), que se puede codificar en una cadena binaria cuya longitud *L(M)* sería mínima en el caso de tener la partición óptima de la red. Esto se puede lograr mediante la aplicación MapEquation, la cual aprovecha la dualidad entre encontrar la mejor estructura modular en una red y minimizar la longitud de la descripción de los movimientos de un caminante aleatorio en dicha red. Es decir, para cada partición modular dada de la red, existe un costo de información asociado con la descripción de los movimientos del caminante aleatorio, o del flujo. Algunas particiones generan longitudes de descripción, unas cortas y otras más largas. La partición con la longitud de descripción más corta es la que mejor captura la estructura modular de la red en cuanto a la dinámica de su flujo de información ^33^^-^^35^.

Dado que la idea era encontrar módulos con distancia semántica mínima, es decir, aquellos correspondientes a los términos más estrechamente relacionados de manera local en redes con miles de aristas, la combinación propia de Infomap, con un algoritmo que minimiza la distancia del código y tiene poca complejidad computacional, lo convirtió en la elección apropiada.

Por último, los módulos se etiquetaron con el nombre del nodo con el mayor índice PageRank (IPR), lo que constituye, de hecho, un tipo de centralidad de valor propio ^35^, medida con base en diversos algoritmos que asignan de forma numérica la relevancia de los documentos indexados por un motor de búsqueda, por lo que las comunidades constituyen unidades semánticas estructuradas de mayor complejidad que los términos MeSH individuales.

## Resultados

### Minería computarizada de los documentos

Se obtuvieron 41 artículos científicos publicados entre el 2011 y el 2018. Los lugares registrados por la adscripción, por lo menos, de un artículo fueron Estados Unidos, Europa, China, Brasil, México, Canadá y Sudáfrica.

### Red principal

**Figura 2:**
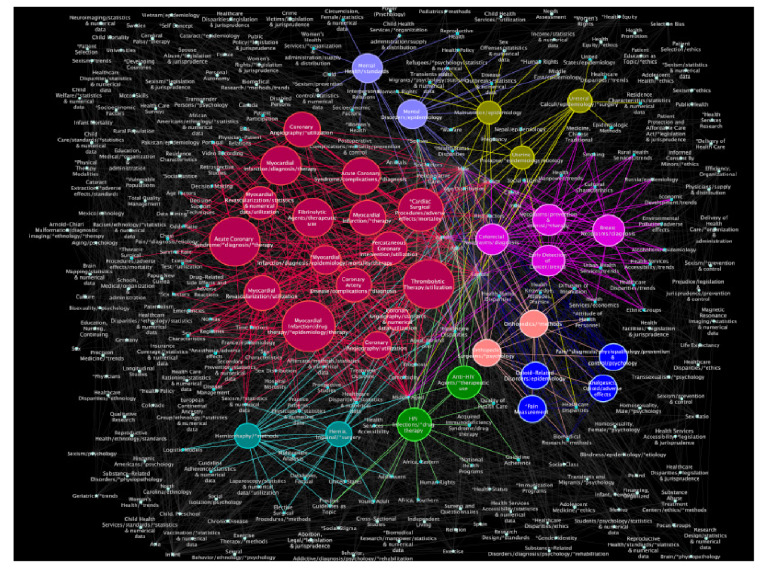
Red principal. Se resaltan los nodos relacionados con algunas enfermedades crónicas, los cuales aparecen con tamaño aumentado y colores brillantes para su mejor distinción.

Las principales características topológicas de las redes analizadas se muestran en el cuadro 1. La estructura revela una red principal con 301 nodos (o términos) y 3.365 enlaces (o relaciones). Se pudo detectar en ella una aparente tendencia a priorizar la investigación relacionada con las disparidades de sexo y algunas enfermedades, específicamente aquellas que involucran la enfermedad cardiovascular, como se aprecia en los 16 nodos de color rojo de la figura 2. En color rosa, se ven los nodos relacionados con tumores o cáncer de mama o colorrectales (4 nodos y 108 centralidades de grado acumulados); en azul, los relacionados con el dolor y las propiedades de analgesia o narcosis, así como los relacionados con la salud y la enfermedad mental (6 nodos y 68 centralidades de grado acumulados); en verde, los relacionados con las infecciones por el virus de inmunodeficiencia humana (3 nodos y 46 centralidades de grado acumulados), y con menor tamaño se aprecian los nodos en color naranja, amarillo y turquesa relacionados con otros padecimientos o métodos médicos, quirúrgicos y ortopédicos, hernias, desnutrición, prolapso uterino y cálculos uretrales.

**Cuadro 1:** Características topológicas de las redes analizadas

Red	Nodos	Aristas	Coeficiente de agrupamiento	Longitud de camino	Centralidad	Número promedio de vecinos	Densidad de red
Principal	301	3.365	0,910	1,962	0,851	22,359	0,075
EVC	205	518	0,194	2,766	0,480	5,054	0,025
Mujeres	277	3.154	0,907	1,917	0,924	22,773	0,083
Hombres	237	2.834	0,902	1,899	0,906	23,916	0,101
Emergente	40	135	0,95	1,0	0,061	6,75	0,173

EVC: enfermedad cardiovascular

En la red principal también se aprecian algunos términos cercanos o primeros vecinos asociados con los nodos de los padecimientos y con la calidad de la atención. Por ejemplo, cerca de los nodos en color rojo (enfermedad cardiovascular), se encontraron términos no esperados sobre la relación entre médico y paciente, la adopción de decisiones médicas y el manejo de la enfermedad; por otro lado, también se encontraron términos más comúnmente asociados con la supervivencia, los factores de riesgo y los estilos de vida. Cerca de los de color rosa, se pueden apreciar términos que, aparentemente, están poco vinculados con los tumores o el cáncer, tales como estigma social, características culturales y acceso a los servicios de salud, y cerca de los azules, relacionados con dolor y analgesia, se aprecian términos como salud de la mujer, servicios organizados de atención en salud para mujeres, ofensas sexuales y política de salud, entre otros.

En los cuadros 2 y 3, se presentan los resultados de la comparación de los valores de las centralidades analizadas en la red principal al considerar los MeSH correspondientes a condiciones médicas patológicas. Por ejemplo, en el cuadro 2 se aprecia que, aunque hubo diferencias en la distribución de rango de las diferentes medidas de centralidad usadas (centralidad de grado, centralidad de intermediación, centralidad de cercanía), la tendencia reportada con respecto a los padecimientos cardiovasculares se mantuvo.

**Cuadro 2:** Comparación de las diferentes medidas de centralidad para los nodos asociados con condiciones médicas relacionadas con la enfermedad cardiovascular

Términos MeSH	CVP	CG	PCMC	CI	CC	CA
Fibrinolytic Agents/therapeutic use	0,056516256	23	1,92857143	0	0,51851852	1
Myocardial Infarction/drug therapy/^*^epidemiology/therapy	0,056516256	23	1,92857143	0	0,51851852	1
Myocardial Revascularization/utilization	0,056516256	23	1,92857143	0	0,51851852	1
Thrombolytic Therapy/utilization	0,056516256	23	1,92857143	0	0,51851852	1
"Myocardial Infarction/diagnosis/epidemiology/mortality/therapy	0,04928961	18	1,93877551	0	0,51578947	1
Coronary Angiography/utilization	0,049289722	18	1,93877551	0	0,51578947	1
Percutaneous Coronary Intervention/utilization	0,04928957	18	1,93877551	0	0,51578947	1
Acute Coronary Syndrome/complications/^*^diagnosis	0,04531952	17	1,97619048	0	0,5060241	1
Chest Pain/^*^diagnosis/etiology	0,04531952	17	1,97619048	0	0,5060241	1
Coronary Angiography/^*^utilization	0,04531952	17	1,97619048	0	0,5060241	1
Coronary Artery Disease/complications/^*^diagnosis	0,04531952	17	1,97619048	0	0,5060241	1
"Cardiac Surgical Procedures/adverse effects/mortality	0,036228206	15	1,95578231	0	0,51130435	1
Acute Coronary Syndrome/^*^diagnosis/^*^therapy	0,04339972	15	1,9829932	0	0,50428816	1
Coronary Angiography/statistics & numerical data/utilization	0,043399736	15	1,9829932	0	0,50428816	1
Myocardial Infarction/diagnosis/therapy	0,043399762	15	1,9829932	0	0,50428816	1
Myocardial Revascularization/statistics & numerical data/utilization 0,04339972	0,04339972	15	1,9829932	0	0,50428816	1
Myocardial Infarction/^*^therapy	0,007807993	2	2,38435374	0	0,41940086	1

CVP: centralidad de valor propio; CG: centralidad de grado; PCMC: promedio de caminos más cortos; CI: centralidad de intermediación; CC: centralidad de cercanía; CA: coeficiente de agrupamiento.

**Cuadro 3:** Comparación de las diferentes medidas de centralidad para los nodos asociados con condiciones médicas relacionados con otras enfermedades

Términos MeSH	CVP	CG	PCMC	CI	CC	CA
Breast Neoplasms/diagnosis	0,06038379	26	1,91836735	0	0,5212766	1
Colorectal Neoplasms/diagnosis	0,060383793	26	1,91836735	0	0,5212766	1
Neoplasms/prevention & control/^*^therapy	0,06038379	26	1,91836735	0	0,5212766	1
Hernia, Inguinal/^*^surgery	0,05517667	20	1,96598639	0	0,50865052	1
Arnold-Chiari Malformation/diagnostic imaging/^*^ethnology/^*^therapy	0,044969346	19	2,10544218	0	0,47495961	1
HIV lnfections/^*^drug therapy	0,042333294	19	1,94217687	1.51E-04	0,51488616	0.74269006
^*^Pain Measurement	0,030480828	16	1,97278912	0	0,50689655	1
Opioid-Related Disorders/epidemiology	0,030480828	16	1,97278912	0	0,50689655	1
Orthopedics/^*^methods	0,030480828	16	1,97278912	0	0,50689655	1
Pain/^*^diagnosis/physiopathology/prevention & control/psychology	0,030480828	16	1,97278912	0	0,50689655	1
Malnutrition/epidemiology	0,029936733	15	1,95578231	0	0,51130435	1
Mental Disorders/epidemiology	0,029936733	15	1,95578231	0	0,51130435	1
Cerebral Palsy/^*^therapy	0,034478564	14	1,95238095	0	0,51219512	1
Acquired Immunodeficiency Syndrome/drug therapy	0,03175653	13	1,96258503	0	0,50953206	1
Blindness/epidemiology/^*^etiology	0,02833061	12	1,95918367	0	0,51041667	1
Cataract Extraction/^*^adverse effects/standards	0,02833061	12	1,95918367	0	0,51041667	1
Cataract/^*^epidemiology	0,02833061	12	1,95918367	0	0,51041667	1
Disabled Persons	0,015048198	12	2,33333333	0	0,42857143	1
Ureteral Calculi/epidemiology/^*^surgery	0,04004379	12	1,96598639	0	0,50865052	1
Substance-Related Disorders/^*^physiopathology	0,022431524	11	2,02380952	0	0,49411765	1
Uterine Prolapse/^*^epidemiology/etiology	0,011812069	5	2,03741497	0	0,49081803	1

CVP: centralidad de valor propio; CG: centralidad de grado; PCMC: promedio de caminos más cortos; CI: centralidad de intermediación; CC: centralidad de cercanía;.CA: coeficiente de agrupamiento

En el cuadro 3 se evidencia una excepción notable e interesante por sus posibles connotaciones; es el caso del HIV, cuyo rango en las distribuciones ordenadas por centralidad de intermediación fue bastante menor, es decir, de mayor importancia, que su rango en las distribuciones ordenadas por centralidad de grado o de cercanía.

### Red secundaria de enfermedad cardiovascular

**Figura 3:**
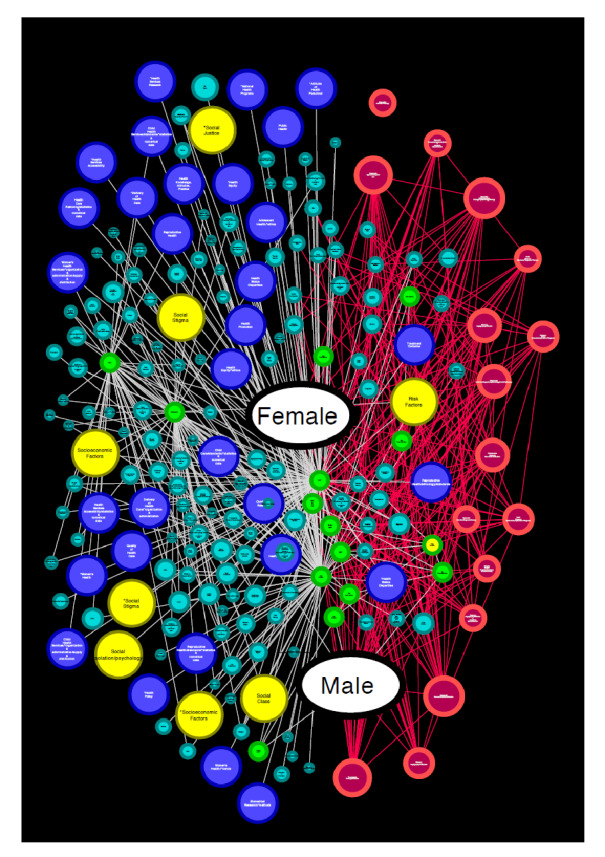
Red de enfermedades cardiacas. En esta red se aprecia la agrupación por nodos con colores ordenados para mostrar la conexión indirecta de los términos MeSH relacionados con los padecimientos cardiacos (en rojo) y aquellos que hacen referencia a términos de factores físicos, del sistema de salud, de los factores sociales determinantes y de otros (en colores azul claro, azul oscuro, amarillo o verde).

Como se aprecia en la figura 3, en el caso de las enfermedades cardiovasculares (nodos en color rojo), se obtuvo una red secundaria con 205 nodos y 518 enlaces, que incluye información tanto para el término "femenino" *(female)* (centralidad de grado: 17) como para "masculino" *(male)* (centralidad de grado: 18), en los que no se aprecia una tendencia de conexión particular entre la enfermedad y el sexo.

Por otro lado, se aprecia un patrón de conexión indirecta, cada vez con una centralidad de grado menor entre los nodos rojos y los de color azul claro, azul oscuro, amarillo y verde, los cuales aluden a los factores de infancia, adolescencia, expectativa de vida, clase social, religión, derechos de las mujeres, derechos humanos, factores socioeconómicos, acceso a servicios de salud, acceso a servicios de salud de mujeres, estigma social, justicia social, características residenciales, políticas de salud, medicina de precisión, investigación biomédica y difusión de innovación.

Esta forma de relación indirecta sugiere una brecha entre la información sobre la enfermedad cardiovascular y otros factores que pueden incidir o modificar su manejo médico, como la etiología y el diagnóstico del dolor de pecho, la relación entre médico y paciente, la participación del paciente en lo relacionado con su salud, cuidados posquirúrgicos, manejo de la enfermedad, mortalidad hospitalaria, emergencias, pronóstico, tasa de supervivencia, prevención secundaria, cuidados preoperatorios, complicaciones posoperatorias y efectos adversos.

### Red emergente de la diferencia entre mujeres y hombres

**Figura 4:**
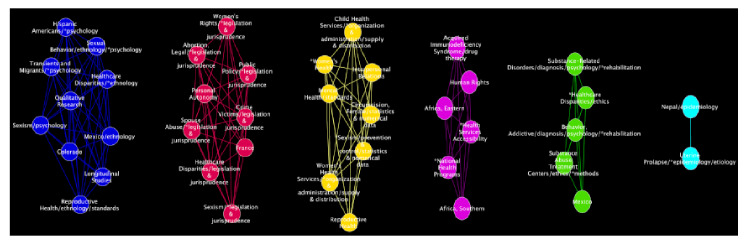
Red emergente. Esta red agrupa los nodos por colores y centralidades de grado (CG). Se reconstruyó a partir de la diferencia de nodos y vecinos de las redes secundarias de hombres y mujeres para conocer los términos incluidos en una y no en la otra según se aprecian en esta figura.

Del análisis de las redes semánticas secundarias se desprende que la red de mujeres tenía 277 nodos y 3.154 enlaces, y la de hombres, 237 nodos y 2.834 enlaces. Se determinaron, además, los términos que se encontraban en la red de mujeres y no en la de hombres, lo que evidenció una estructura con seis componentes compuestos de 40 nodos y 135 enlaces (figura 4).

El componente de color azul con 9 centralidades de grado incluyó algunos términos relacionados con las diferencias culturales, los migrantes y las disparidades en el cuidado de la salud. En rojo, con 8 centralidades de grado, aparecieron términos sobre los derechos de las mujeres, la política pública y las disparidades en salud. En amarillo, con 7 centralidades de grado, se registraron términos relacionados con los servicios de salud para mujeres y la circuncisión femenina. En morado, con 5 centralidades de grado, aparecieron aquellos términos relacionados con África, el síndrome de inmunodeficiencia adquirida y algunos componentes del sistema nacional de atención médica y el acceso a él. En verde, con 4 centralidades de grado, se aprecian los términos relacionados con los centros de salud que brindan terapia o rehabilitación a personas dependientes de sustancias, México, y disparidades en el cuidado de la salud. Por último, en azul cian, con 1 centralidad de grado, solo hay dos términos: Nepal y prolapso uterino, lo que refleja su poca importancia por el reducido grado de conexión.

### Modularidad

**Figura 5:**
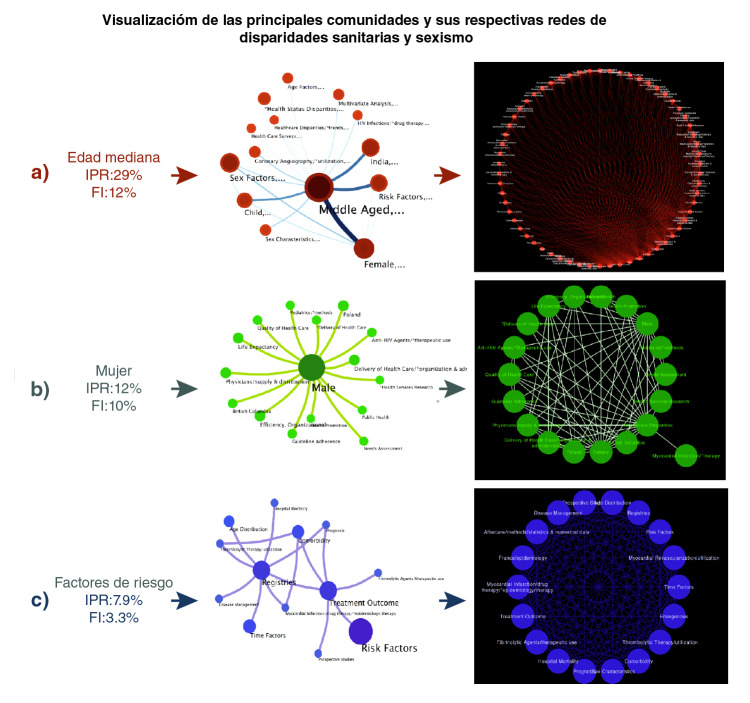
Visualización modular de las principales comunidades y sus respectivas redes. Los círculos más grandes representan las comunidades que comparten más enlaces de información entre nodos. El nombre de las etiquetas de las comunidades corresponde al del nodo con el mayor PageRank.

En la figura 5 se presentan los nodos intraconectados representativos de los MeSH. Se aprecian las comunidades de aquellos términos relacionados principalmente con factores de riesgo. Los enlaces y su grosor simbolizan el flujo de información (concurrencia) entre nodos. Las características topológicas de las redes secundarias correspondientes a la partición modular en comunidades se presentan en el cuadro 4.

**Cuadro 4:** Características topológicas de las redes secundarias correspondientes a la partición en comunidades

Red	Nodos	Aristas	Coeficiente de agrupamiento	Longitud de camino	Centralidad	Número promedio de vecinos	Densidad de red
Edad mediana	62	552	0,881	1,723	0,630	17,806	0,292
Mujer	19	67	0,848	1,620	0,618	7,053	0,392
Factores de riesgo	18	153	1,0	1,0	0,0	17	1,0

El componente más conectado en la figura 5a fue la "edad mediana" con un IPR de 29 % y un flujo de información (FI) de 12 % (debe recordarse que, cuanto mayor el flujo de información de un módulo, mayor la coherencia interna de sus nodos, y que la suma de la información total en las redes implica que FI=1,0). Se registraron 62 nodos que representan los términos más relacionados con la edad y los factores socioeconómicos, la accesibilidad a los servicios de salud, los patrones de práctica médica, y otros conceptos relativos a la planificación de la asignación equitativa de los recursos de salud disponibles. Es interesante señalar que los patrones de conectividad de los nodos de una comunidad son característicos. Para hacerlos explícitos, se determinó un conjunto de medidas de centralidad y otros atributos topológicos de los nodos en el contexto de sus respectivas comunidades (cuadro suplementario 2).

En la figura 5b se muestra la comunidad asociada con la condición de ser mujer (IPR=12 % y FI=10 %). Algunos de los 19 términos más relacionados con esta son: disparidad en salud, esperanza de vida, atención médica del parto, calidad de la asistencia sanitaria y HIV, entre otros (cuadro suplementario 3).

En la figura 5c se presenta el módulo asociado con los factores de riesgo (IPR=7,9 % y FI=3,3 %) y 18 conceptos relacionados con la justicia social, la identidad de género, los derechos de las mujeres, el paternalismo, y la cultura, entre otros. Resulta interesante el papel poco destacado de estos temas en la literatura biomédica analizada (cuadro suplementario 4).

## Discusión

### Minería computarizada de términos MeSH

En concordancia con lo reportado en otros estudios, la minería computarizada tal como se empleó aquí ofrece métodos poderosos, rápidos y de bajo costo para la exploración masiva de textos científicos. La utilización de la ontología MeSH en lugar del texto completo no solo reduce el tiempo de cálculo matemático en algoritmos de concurrencia, sino que permite un mayor rendimiento en el análisis del conjunto de datos. Si se exploran adecuadamente, los términos MeSH permiten extraer con precisión características representativas de grandes cantidades de datos ^36^.

El procesamiento de un corpus de texto simple a un lenguaje natural respalda el diseño de mecanismos eficaces de comunicación computacional que son interpretados por programas que simulan el contenido semántico de los conceptos y favorecen la construcción de mapas de conectividad ontológica ^37^. En este estudio sobre disparidades en salud, se construyeron redes semánticas para inferir la forma en que se reporta la información en algunos documentos científicos.

### Construcción de redes semánticas primarias y secundarias

Como ya se mencionó en la introducción, las causas de las disparidades corresponden a múltiples factores ^38^. En la enfermedad cardiovascular, por ejemplo, aunque estas disparidades se han asociado con la atención médica y el manejo clínico, existen otros elementos que interactúan y pueden modificar la expresión de la enfermedad ^39^^-^^42^. Se ha reportado, asimismo, que las estructuras socioeconómicas y los sistemas sanitarios son responsables de algunas disparidades debidas al sexo en varios sentidos: al mostrar apatía para incidir y controlar factores de riesgo modificables, por la indiferencia en el reconocimiento de signos y síntomas, la insensibilidad frente al acceso y el uso de tratamientos químicos o quirúrgicos de última generación, o el desinterés por ayudar a la recuperación, pero, sobre todo, el rezago en la producción de información comprobada sobre el papel del sexo en la adopción de las decisiones clínicas y el desarrollo de políticas de mejoramiento de la calidad y la eficiencia de la atención cardiovascular de las mujeres ^43^^,^^44^.

Lo que se observó en la red principal sobre disparidades en salud y sexismo evidencia que los MeSH más frecuentes relativos a las condiciones médicas, se relacionaban con la enfermedad cardiovascular. Se determinaron 17 nodos, entre ellos, los siguientes MeSH: *Cardiac Surgical Procedures, Myocardial Infarction, Acute Coronary Syndrome, Coronary Angiography, Coronary Artery Disease, Fibrinolytic Agents, Myocardial Revascularization, Percutaneous Coronary Intervention* y *Thrombolytic Therapy.*

Estos hallazgos se esperaban dado el incremento de las publicaciones científicas relacionadas con la enfermedad cardiovascular y el sexo femenino, especialmente porque la enfermedad coronaria, tradicionalmente considerada una enfermedad masculina, se ha convertido en una gran amenaza biológica, hormonal y genética para las mujeres ^45^^,^^46^.

En las últimas dos décadas, la investigación centrada en mujeres con riesgo de enfermedad isquémica del corazón ha ayudado a aclarar algunos de los factores específicos del sexo importantes en la prevención y detección temprana de disparidades en salud, especialmente en aquellas mujeres que están en desventaja social debido a su nivel de ingresos, su nivel educativo, y los factores ambientales y residenciales ^43^.

Por otro lado, la disparidad en la mortalidad por enfermedades cardiovasculares entre mujeres y hombres también ha impulsado la investigación específica por sexo, así como el desarrollo de directrices específicas y el inicio de campañas de sensibilización en Estados Unidos, tanto, que se reportó una sensible disminución del 39 % en las tasas de mortalidad por dichas enfermedades en las mujeres entre 1999 y 2011. A pesar de esta disminución, la carga de la enfermedad, la coexistencia con otras enfermedades y la prevalencia de los factores de riesgo siguen siendo altas ^43^.

Con el ejemplo de la red de MeSH relacionados con la enfermedad cardiovascular, se evidenció una aparente disonancia entre aquellos relacionados con la enfermedad y los que describen los factores sociales determinantes, los factores de riesgo, la edad, la actividad física y algunas comorbilidades. Este hecho lleva a pensar que la información sobre disparidades entre los factores biomédicos y el contexto social, continúa presentando vacíos, por lo menos en materia de evidencia científica ^43^^,^^47^.

Como se pudo apreciar tanto en la red principal de enfermedad cardiovascular como en la secundaria, coexisten términos asociados con otras enfermedades crónicas también relacionadas con las disparidades de sexo, lo que se suma a la creciente evidencia científica que sugiere que el sexo es un factor independiente que influye en la prevalencia de enfermedades como el cáncer, las enfermedades mentales, el HIV y el sida, así como en los efectos de los medicamentos, los efectos adversos y el manejo del dolor ^48^^,^^49^.

En cuanto a los factores de riesgo, se ha demostrado que, aunque los hombres y las mujeres comparten algunas características que aumentan su probabilidad de sufrir una enfermedad coronaria (tabaquismo, colesterol elevado, diabetes, estilo de vida sedentario, hipertensión, obesidad), varias de ellas son más agudas en las mujeres ^50^. Además, ciertos factores de riesgo específicos del sexo, como el inicio temprano de la menopausia, enfermedades inflamatorias como el lupus y la artritis reumatoide, y complicaciones del embarazo como la preeclampsia y la diabetes gestacional, se asocian con una mayor incidencia de enfermedades del corazón ^51^. Hasta el 50 % de la reducción de la mortalidad por enfermedades cardiovasculares se puede atribuir a la reducción de estos factores de riesgo, aunque el esfuerzo de sensibilización y detección temprana debe ser continuo si se quiere seguir reduciendo las tasas de mortalidad ^43^.

Los hallazgos de la red emergente de términos que solo se encontraron en la red secundaria de mujeres y no en la de hombres, confirma lo reportado en la literatura especializada: que la salud y el bienestar de las mujeres responden a una combinación de políticas de atención médica que inciden en el tipo de cobertura de los planes de salud y el acceso a servicios preventivos, de detección y tratamiento ^52^^-^^55^. Los resultados se ajustan parcialmente a los postulados del modelo biopsicosocial según el cual las enfermedades crónicas responden a factores biológicos, pero también, a las condiciones psicológicas y sociales ^56^.

Por otro lado, aunque los nodos de color azul cian de la red emergente parecen ser de poca importancia, el problema que evidencian no es menor. La organización de derechos humanos Amnistía Internacional ha señalado que alrededor del 10 % de las mujeres en Nepal ha experimentado un prolapso uterino debido a la discriminación de sexo. Además, estas mujeres cargan un fuerte estigma social y son señaladas como perezosas cuando están demasiado enfermas para trabajar, en pocas palabras, otra forma de disparidad en el cuidado de la salud ^57^.

### Modularidad

En cuanto al análisis topológico de la modularidad mediante el algoritmo Infomap, este permitió organizar muchos nodos según su distribución estructural en cada comunidad. Si bien la comunidad más conectada, la de "edad mediana" (IPR=29 % y FI=12 %), contenía dos de los principales términos relacionados con los factores de riesgo (edad y aspectos socioeconómicos), los términos relacionados con la calidad de la atención se destacaron más con este análisis que en el resto de las comunidades, por ejemplo, los relativos a la asignación equitativa de los recursos de salud disponibles, la justicia social y los derechos de las mujeres. Debe señalarse que, al igual que en la red principal, este fenómeno no solo se presenta asociado con los padecimientos cardiacos, sino también con otras comorbilidades.

Estos resultados concuerdan con lo informado en la literatura sobre los factores que influyen o modifican la calidad de la atención y las disparidades por sexo, entre los que se incluyen la mala comunicación entre paciente y médico, la cultura, las opiniones holísticas de la mente y el cuerpo, la priorización del manejo de los síntomas frente a la cura de la enfermedad y la participación de la familia en la adopción de decisiones médicas ^52^. Asimismo, los factores internos y externos del sistema de prestación de servicios de salud, como la falta de acceso a la atención médica oportuna y la escasa protección financiera, también pueden incidir en su calidad ^53^^,^^54^.

En cuanto a la comunidad relativa a la condición de ser mujer, también se observó una relación con términos sobre la calidad asistencial y el HIV, por ejemplo. Desde la aparición del HIV-sida la discriminación en los sistemas de salud sigue estando en el centro de las experiencias negativas de las personas con el virus ^58^. Por otro lado, sabemos que los síntomas clínicos y las complicaciones del HIV que experimentan las mujeres son diferentes a las de los hombres. Aun así, en una revisión del 2016 sobre la inclusión de las mujeres en las investigaciones sobre el HIV en los Estados Unidos, se encontró que estas solo representaban el 19,2 % de los participantes en los estudios antirretrovirales, el 38,1 % en los estudios de vacunación y el 11,1 % en aquellos que exploraban tratamientos ^59^^,^^60^. Este es otro ejemplo de disparidad en la atención en salud por actitudes sexistas, que se hizo incluso más evidente en el análisis de comunidades de términos MeSH adelantado en el presente estudio.

En conclusión, si se quiere contribuir a corregir las disparidades en salud, deben explorarse integralmente los mecanismos sociales, biológicos y clínicos que afectan a hombres y mujeres. Aunque las diferencias sexuales están cada vez mejor documentadas en la literatura biomédica, como se evidencia en los artículos sobre la prevalencia de los factores de riesgo cardiovascular, las condiciones fisiológicas y psicosociales, así como las manifestaciones clínicas y la incidencia de la enfermedad, los médicos insisten en referirse a la atención médica de la mujer como "medicina de bikini" asumiendo que pueden diagnosticar y tratar a ambos sexos de la misma manera ^61^. Esta tendencia también se percibe como una manera de perpetuar la disparidad en la calidad de la atención médica.

### Acerca del análisis de la literatura asistido por computador

Dada la abrumadora cantidad de información disponible en las bases biomédicas de datos, es invaluable contar con un recurso que lea automáticamente la información y genere patrones de visualización de los conceptos y las asociaciones, especialmente para los responsables de las decisiones. En este sentido, el presente estudio demuestra la utilidad de un novedoso método basado en la búsqueda rápida y masiva de evidencia científica mediante redes semánticas de términos MeSH.

En la práctica, la determinación de módulos o comunidades es fundamental para inferir relaciones entre los MeSH a partir de la topología de redes semánticas, y puede ser una guía confiable para futuros estudios sobre disparidades en salud y sexismo, así como para profundizar en sus implicaciones teóricas. Los análisis hechos aquí revelaron algunos aspectos esenciales del fenómeno estudiado que, aparentemente, se han pasado por alto de manera reiterada, lo que ha profundizado aún más la brecha que hay que cerrar para pasar de la discusión de estas desigualdades a la formulación de políticas de salud pública para atenderlas a partir de un enfoque social centrado en el individuo.

Puede decirse que la investigación que examina la información relacionada con los dos sexos en su contexto social amplía la posibilidad de extrapolar los resultados y de aplicarlos en el ámbito médico y clínico ^11^. Lo contrario llevaría a interpretaciones erróneas en torno a las decisiones y los comportamientos sociales frente a la salud y la enfermedad, con consecuencias negativas para la provisión de los servicios y la formulación de políticas de salud e, incuso, para la lucha contra estructuras de poder (incluidas las jerarquías del conocimiento), que perpetúan la inequidad y la mala salud ^6^^,^^9^^,^^12^.
